# Personality traits and symptoms of anxiety and depression in patients with primary vitreous floaters

**DOI:** 10.1007/s00417-024-06477-y

**Published:** 2024-05-03

**Authors:** Hugo Senra, Zaria Ali, Tariq Aslam, Niall Patton

**Affiliations:** 1 Institute of Electronics and Informatics Engineering of Aveiro (IEETA), University of Aveiro, Aveiro, Portugal; 2 School of Health and Social Care, University of Essex, Colchester, UK; 3 Manchester Royal Eye Hospital, Manchester, UK; 4 University of Manchester, Manchester, UK

**Keywords:** Vitreous opacities, Personality traits, Well-being, Depression, Anxiety

## Abstract

**Purpose:**

We investigated personality traits and symptoms of anxiety and depression in patients with primary vitreous floaters.

**Methods:**

A U.K. sample of adult patients (> 18 years old) with vitreous floaters of a minimum of three months severe enough to seek a consultation was assessed for personality traits (The Big Five Inventory (BFI)), symptoms of depression (Patient Health Questionnaire-9), and symptoms of anxiety (Generalized Anxiety Disorder Questionnaire-7).

**Results:**

149 patients participated in the study. Compared to the general population, our sample had a significantly increased score in the domain of BFI-neuroticism (3.27 vs 2.97, *ρ* < 0.0001, d = 0.38) and reduced score in the domain of extraversion (2.97 vs 3.24, *ρ* < 0.0001, d = 0.33). Female patients scored significantly higher than male patients on BFI-neuroticism (*ρ* = 0.01), and on BFI-agreeableness (*ρ* = 0.01). Age was positively correlated with BFI-Conscientiousness (r = 0.19, *ρ* = 0.02) and with BFI-Agreeableness (r = 0.20, *ρ* = 0.01). 36% of our sample had moderate to severe symptoms of depression, and 43% had moderate to severe symptoms of anxiety.

**Conclusions:**

Our study highlights the underlying psychological traits of patients with severe vitreous floaters and particular mental health needs that deserve further consideration by ophthalmological and vision science clinicians.

**Supplementary Information:**

The online version contains supplementary material available at 10.1007/s00417-024-06477-y.


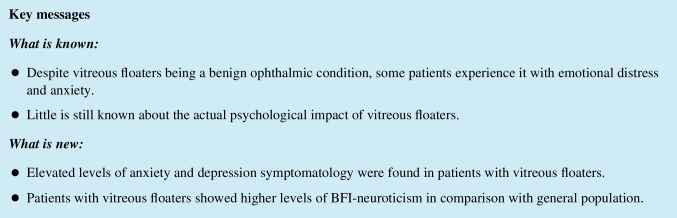


## Introduction

Vitreous floaters are a relatively common ophthalmic symptom resulting from opacities within the vitreous body and may be present in up to 77% of the population [1, 2]. The majority of patients adapt to floaters and are not unduly troubled by their symptoms and do not seek any consultation or therapeutic intervention [3–5]. However, for a small minority, floaters can have a profoundly negative impact on quality of life (vision degrading myodesopsia), and these patients may be less able to adapt to their visual symptoms and more likely seek a consultation for possible therapeutic intervention in the form of a pars plana vitrectomy, despite the potential risks inherent in this intervention.

Although there are studies assessing the quality of life and depressive state of patients with vitreous floaters, there have yet to be any studies assessing underlying personality traits [6]. Personality traits are conceptualised as an individual’s unique adjustment to life, including interests, drives, values, self-concept, abilities and emotional patterns [7]. They are relatively stable measures of human behaviour, although there is evidence that some traits can change across the life span [7]. Identification of these traits may help clinicians recognise patients who are more likely to experience reduced quality of life or be at risk of depression from vitreous floaters which in turn could help identify potential non-surgical therapeutic approaches to offer these patients [4, 6, 8].

We collected data regarding personality traits in patients with primary vitreous floaters, who were sufficiently troubled that they sought consultation regarding their symptoms and/or therapeutic intervention. We analysed our sample for personality traits, based on the Big Five Factors of personality framework [9, 10]. In addition, we investigated the relationship between gender, age, self-reported history of anxiety or depression and previous treatment for floaters on personality traits as well as measures of symptoms of current anxiety and depression in our sample.

## Methods

### Ethics statement

Ethical clearance was granted by the NHS-England Research Ethics Committee (REC reference number 21/PR/1631, IRAS project ID 301344). All research procedures were performed in accordance with relevant guidelines and regulations, and in accordance with the Declaration of Helsinki and in accordance with ICH Guidelines for Good Clinical Practice (CPMP/ICH/135/95) [11]. Informed consent was obtained from all participants in this study.

### Sample

A U.K. sample composed of patients aged 18 or over who have contacted an ophthalmologist to request a consultation regarding treatment for floaters and have had vitreous floaters for a minimum of three months were invited to participate in our study via Manchester Royal Eye Hospital. All participants were from England. The study was advertised to the general public online and via flyers which were distributed to local optometrists and ophthalmologists. A minimum of three months symptom duration was used to ensure the floaters didn’t represent a recent acute posterior vitreous detachment (PVD), whose symptoms often resolve quickly without the need for any intervention and avoid including patients whose floaters are transient and not affecting their quality of life. Patients with the following conditions were excluded: co-existing ocular pathology e.g. uveitis, glaucoma, pathological myopia (refractive error > -6D); known cognitive impairment or learning difficulties that would affect their ability to access and complete the questionnaire; known comorbid psychiatric disorders, including personality disorders; and patients who were not able to read, speak and understand English so they could not comprehend the patient information sheet and questionnaire. After consenting to participate in the study, an online questionnaire was provided. This online questionnaire included demographic and clinic information, such as age, gender, history of mental health problems, previous treatment for mental health problems, and previous treatment for floaters, as well as standardized measures of personality traits, symptoms of depression and symptoms of anxiety.

### Measures

Personality traits were assessed with the ‘Big Five Inventory’ (BFI), a widely used and validated personality questionnaire [9]. The BFI is a 44-item instrument measuring the Big Five Factors of personality [10]: neuroticism, extraversion, agreeableness, conscientiousness, and openness. Neuroticism is characterized by a tendency for negative emotionality, upsetability, poor coping, and poor reactions to illness and to job changes. Extraversion is characterized by energy, enthusiasm, positive emotionality, talkativeness, assertiveness, and social adjustment. Agreeableness is characterized by traits such as altruism, tendermindedness, cooperativeness, trust and tendency for emphasizing good qualities of other people. Conscientiousness is characterized by responsibility, dependability, thinking before acting behaviours, organizing and prioritizing tasks. Openness is characterized by originality, curiosity, intellectualism, independence of mind, and reflection.

The five personality traits are scored via answering a number of statements within each domain. The degree to which patients agree with each statement calculates their score, with certain statements being reverse scored. Each statement is scored on a Likert scale (disagree strongly, disagree a little, neither agree nor disagree, agree a little, strongly agree). In items scored normally disagree strongly will give the minimum score of 1, with strongly agreeing to give a score of 5. For items which are reversed scored disagree strongly will give a score of 5, and strongly agreeing will give a score of 1.

Symptoms of depression were assessed using the Patient Health Questionnaire (PHQ-9) [12]. It consists of 9 questions with four level Likert scale answers (0 [not et al.] to 3 [nearly every day]) providing a total score of 0–27. A score of 0–4 usually indicates the patient is not depressed. A score of 5–9 may indicate mild depression. A score of 10–19 may indicate moderate depression. A score of 20 or more may indicate severe depression.

Symptoms of anxiety were assessed using the Generalized Anxiety Disorder Questionnaire (GAD-7) [13], similar to PHQ-9 with 7 questions with four level Likert scale answers (0 [not et al.] to 3 [nearly every day]), providing a total score of 0–21. A score of 0–5 usually indicates the patient does not suffer from anxiety. A score of 6–9 may indicate mild anxiety. A score of 10 or more could indicate moderate to severe anxiety. Both the PHQ-9 and GAD-7 are widely used in the U.K. and have been validated to use in the monitoring and screening of depression and anxiety [14–17]. Both tools are recommended by NICE to use in the assessment of depression and anxiety [18, 19].

### Data analysis

Statistical analysis was undertaken with R. Sample size calculation for personality traits being significantly different from normal population values, using a one-sample Z-test, determined that with a type I error rate (alpha) set at 0.05 (two-tailed), a power of 0.95 and an effect size of 0.5, a minimum of 51 participants were required. To increase the chances of having a normally distributed sample, to allow us to compare our outcome measures against the normal population mean, we recruited a sample of 153 patients. One-sample Z-tests were used to compare our sample mean scores on each BFI factor against the U.K. normal population mean scores on each BFI factor [20]. The U.K. normative data used for all BFI personality traits were extracted from a large-scale study conducted with about 400 thousand British residents from all U.K. regions, stratified for gender, age, ethnicity, education, employment status, annual personal income, and country of residence (England, Scotland, Wales) [20]. In this study 86% of the participants were from England, although the confirmatory factor analysis (CFI) showed no significant regional differences (no CFI differences of 0.01 or greater). In the same study, the following U.K. normative data were found for each BFI personality trait. BFI-Extraversion: mean = 3.24; sd = 0.82; α = 0.86; BFI-Agreeableness: mean = 3.74; sd = 0.62; α = 0.77; BFI-Conscientiousness: mean = 3.65; sd = 0.70; α = 0.83; BFI-Neuroticism: mean = 2.97; sd = 0.81; α = 0.83; BFI-Openness: mean = 3.67; sd = 0.64; α = 0.79. Age was negatively correlated with BFI traits of Extraversion (r = -0.20), Neuroticism (r = -0.22), and Openness (r = -0.31), and positively correlated with Conscientiousness (r = 0.60), and Agreeableness (r = 0.47). Being female was positively correlated with Agreeableness (r = 0.47). The study did not provide results on mean BFI scores according to gender and age.

Extreme outliers (N = 4) were identified and removed to reduce skewness detected in the dependent variables (BFI factors) and meet the Z-test assumption of normal distribution, with Shapiro–Wilk test reporting a p-value > 0.05 (see Shapiro–Wilk normality tests and corresponding histograms in Supplementary material). T-tests were used to compare mean BFI scores within our sample for categorical variables. Cohen’s d effect sizes were computed for z-tests and for t-tests. Cohen’s d effect sizes are classified as small (d = 0.2), medium (d = 0.5) and large (d ≥ 0.8).

## Results

156 patients met the criteria to participate and have agreed to be enrolled in the present study, with 45 patients being excluded for not having met the inclusion criteria. A final sample of 149 patients with a current or previous diagnosis of vitreous floaters who had sought a consultation regarding their symptoms and met the inclusion/ exclusion criteria of the study, participated in the study and completed all questionnaires. Sample mean age was 34.8 years (SD = 11.8, range 18–76 years). There were 108 males (72.4%). 31/149 (20.1%) patients had undergone treatment for floaters, and in 6 of those 31 patients vitrectomy was performed. 7 patients were receiving dietary supplements, 3 patients had undergone laser vitreolysis treatment, and the remaining 15 patients were receiving eye drops (e.g. atropine) and regular medical follow-ups. A descriptive comparison between our sample and the U.K. normative data characteristics for age and gender are presented in Supplementary Table A. In general, our study sample matches the demographics of the UK normative data [20], except for sex, with the majority of our sample being composed of men (72.5%), whereas in the UK population study most individuals were females (64%).

Mean values for the five BFI domains between our sample and the general population are listed in Table 1. Compared to the U.K. normative data [20], our sample had a significantly increased score in the domain of neuroticism (3.27 vs 2.97, *ρ* < 0.0001, d = 0.38), and reduced score in the domain of extraversion (2.97 vs 3.24, *ρ* < 0.0001, d = 0.33). There were no other differences in the remaining personality traits between our sample population and the U.K. general population reference data.

**Table 1:** BFI personality traits in a sample of patients with vitreous floaters and in general population (U.K.)

	BFI-Neuroticism Mean (SD)	BFI-Agreeableness Mean (SD)	BFI-Conscientiousness Mean (SD)	BFI-Extraversion Mean (SD)	BFI-Openness Mean (SD)
All Sample (N = 149)	3.27 (0.78)* d = 0.38^++^	3.69 (0.55) d = 0.08^++^	3.70 (0.60) d = 0.08^++^	2.97 (0.78)* d = 0.33^++^	3.77 (0.51) d = 0.15^++^
UK-Population Study^1^ (N = 386 375)	2.97 (0.81)*	3.74 (0.62)	3.65 (0.70)	3.24 (0.82)*	3.67 (0.64)

^*^ Z-test with p-value < 0.0001 (BFI-Neuroticism: Z = 4.59; BFI-Extraversion: Z = - 4.05); ^++^ Cohen’s d (Effect size)

^1^ Rentfrow PJ, Jokela M, Lamb ME. Regional personality differences in Great Britain. PLoS One. 2015 Mar 24;10(3):e0122245. https://doi.org/10.1371/journal.pone.0122245

Regarding mental health outcomes in our sample, 55.7% had a self-reported history of depression/anxiety. The sample mean scores on PHQ-9 and GAD-7 was 8.03 (SD = 5.94), and 9.17 (SD = 6.05) respectively. 36% of our sample had moderate/severe symptoms of depression (PHQ-9 ≥ 10). 43% of our sample population had moderate/ severe symptoms of anxiety (GAD-7 ≥ 10).

Exploring the factors within our sample population that were associated with individual personality traits and current depression/ anxiety scores, females had significantly higher BFI-neuroticism (t = -2.49, *ρ* = 0.01) and BFI-agreeableness (t = -2.51, *ρ* = 0.01) scores in our sample with no gender significant differences (*ρ* > *0.05)* in the other domains (Table 2). In our sample, those with a history of depression/anxiety had significantly higher BFI-neuroticism scores (t = -4.87, *ρ* < 0.0001), and higher current symptoms of depression (PHQ-9 scores) (t = -4.19, *ρ* < 0.0001) and anxiety (GAD-7 scores) (t = -4.33, *ρ* < 0.0001) (Table 2). Those that had a self-reported history of having had any treatment for floaters had a lower score for BFI openness (t = 2.14, *ρ* = 0.04) (Table 2). Finally, age was positively correlated with BFI-Conscientiousness (r = 0.19, *ρ* = 0.02) and with BFI-Agreeableness (r = 0.20, *ρ* = 0.01) but no other domains of personality traits.

**Table 2:** BFI personality traits and symptoms of depression and anxiety in a sample of patients with vitreous floaters

		BFI-Neuroticism Mean (SD)	BFI-Agreeableness Mean (SD)	BFI-Conscientiousness Mean (SD)	BFI-Extraversion Mean (SD)	BFI-Openness Mean (SD)	PHQ-9 Mean (SD)	GAD-7 Mean (SD)
Gender (N = 149)	Males (N = 108)	3.18 (0.75)*	3.63 (0.56)*	3.67 (0.58)	2.94 (0.69)	3.71 (0.49)	7.93 (5.99)	8.70 (5.81)
	Females (N = 41)	3.53 (0.79)*	3.85 (0.46)*	3.80 (0.64)	3.03 (0.80)	3.90 (0.54)	8.27 (5.85)	10.41 (6.54)
History of Depression or Anxiety (N = 149)	No (N = 66)	2.95 (0.77)**	3.71 (0.51)	3.81 (0.55)	3.08 (0.74)	3.71 (0.49)	5.92 (4.39)**	6.89 (5.47)**
	Yes (N = 83)	3.53 (0.69)**	3.67 (0.57)	3.62 (0.62)	2.87 (0.69)	3.81 (0.52)	9.67 (6.47)**	10.96 (5.90)**
Previous Treatment for Floaters (N = 148)	No (N = 117)	3.27 (0.78)	3.72 (0.53)	3.72 (0.61)	2.94 (0.72)	3.81 (0.51)*	8.28 (5.93)	9.35 (6.02)
	Yes (N = 31)	3.31 (0.81)	3.57 (0.58)	3.65 (0.57)	3.02 (0.72)	3.59 (0.51)*	7.03 (6.03)	8.58 (6.33)

^*^ T-test with p-value < 0.05; **T-test with p-value < 0.0001

## Discussion

The current study explores personality traits associated with patients who are severely affected by vitreous floaters, a still poorly understood ophthalmic condition from a psychological perspective [6]. Our study highlights greater BFI neuroticism traits as well as lower extraversion traits in a sample of patients with vitreous floaters, compared with the normal population, with a medium effect size. These findings might contribute to a better understanding of patients’ behaviours in relation to vitreous floaters. Some patients appear to be more susceptible to experience some form of emotional distress in relation to floaters compared to the majority who find floaters merely an irritation or inconvenience. The understanding of psychological implications of vitreous floaters is important to help clinicians managing symptomatic vitreous floaters and patients’ expectations [4, 6]. For instance, in some cases where the patient is more concerned about the symptomatic floaters and there is no medical evidence supporting patient’s concern, a doctor-patient discussion in the context of the ophthalmology consultation might be helpful to manage patient expectations and anxiety about the symptoms.

The hypothesis that patients with significant vitreous floaters have higher underlying levels of neuroticism may partially explain why some are more worried about floater symptoms, may seek medical treatment, and also why they suffer significant visual effects and profound issues with quality of life despite having a benign non-sight threatening condition [4, 6, 21]. It is known that individuals with high neuroticism traits are more prone to worry about their physical health and to experience physical symptoms with emotional distress [22–25]. It is important to note that all individuals have a degree of neuroticism as part of their personality trait, but those patients with symptomatic floaters appear to have a higher neuroticism scores compared to the general population. Studies on other ophthalmic conditions such as dry eye disease have also found higher levels of neuroticism in these patients [24, 25]. Subjective symptoms of dry eye disease were positively associated with neuroticism [24, 25], isolation tendencies [24], and perceived stress [25] and higher neuroticism traits were associated with reduced quality of life [25]. In addition, the lower extraversion BFI personality trait scores in our sample population is consistent with the elevated neuroticism traits found, as extraversion traits are commonly associated with positive emotional adjustment, energy, and interpersonal interactions which are less common characteristics in people with higher levels of neuroticism [24, 25]. Finally, the emotional distress associated with floaters can also lead to somatization in some patients, which might entail a tendency to experience and communicate somatic distress in response to psychological stress and to seek medical help for it. Our study is, therefore, consistent with previous findings suggesting a link between increased somatization and low extraversion and high neuroticism [26–28].

Though our sample was predominantly male, BFI personality traits showed female patients scoring significantly higher than males for neuroticism and agreeableness traits, which is not completely consistent with the general population findings, where only agreeableness was found significantly positively correlated with females [20]. The ophthalmic literature addressing personality traits is very limited, but the available studies did not find any personality traits significantly associated with gender [24, 25]. It is, therefore, unclear why gender may play a role in psychological measures for vitreous floaters sufferers, with more research needed.

In our study agreeableness and conscientiousness traits were significantly and positively correlated with age, whereas in the general population study investigating BFI personality traits [10, 20], age was negatively correlated with extraversion, neuroticism and openness, and positively correlated with agreeableness, and conscientiousness, further indicating how the underlying psychological traits in this population differs from the general population. Previous studies on personality traits in other ophthalmic conditions such as dry-eye disease have not suggested any significant associations between personality traits and age [24, 25]. As psychological literature addressing personality traits in eye disease patients is scarce, including for vitreous floaters, future studies with larger patient samples will clarify the actual role of demographical data such as age and gender. In our study, a considerable number of patients met the PHQ-9 criteria for moderate to severe symptoms of depression (36%) and anxiety (43%), which is higher when compared with the prevalence found in other studies (25% for depression; 10%-30% for anxiety) with patients with other ophthalmic conditions (e.g. age-related macular degeneration; diabetic retinopathy; dry eye diseases; glaucoma) [29]. Previous research has highlighted a relatively high prevalence of symptoms of anxiety and depression in patients with vitreous floaters [5, 6, 21]. Studies also suggested that treatment for vitreous floaters might significantly reduce symptoms of depression and anxiety and substantially improve quality of life [6, 21]. The available literature on vitreous floaters is sparse regarding the factors underpinning symptoms of depression in this patient group [6]. A cross-sectional study conducted with a small sample (N = 28) of patients with symptomatic vitreous floaters found age to be negatively correlated with symptoms of depression (r = -0.42, *ρ* = 0.025) [21]. Across different ophthalmic conditions, depression has been associated with visual impairment and poor visual function, poor self-reported health, multimorbidity, poor social support, and poor quality of life [30–33]. Finally, the formal diagnosis of clinical depression and anxiety is not made solely on the basis of self-reported questionnaires, with further clinical investigation being needed to come to a final diagnosis. The elevated prevalence of depression and anxiety symptomatology in our sample should, therefore, be interpreted with caution and be taken as into consideration by clinicians when attending vitreous floaters patients showing apprehension about their symptoms.

Although patients who did not receive any treatment for vitreous floaters scored significantly higher on BFI-openness, which is a personality trait known to be associated with curiosity, intellectualism, independence of mind, and reflection, it must be borne in mind that only a small number of our sample received any treatment for floaters and for those that did receive treatment, only 9/31 had what may be considered a definitive treatment for floaters (i.e. surgical vitrectomy or YAG vitreolysis). Therefore, the increased trait of openness may reflect a greater willingness to consider “alternative” treatment modalities in our sample. Though, we divided the population into those having had treatment versus those not receiving treatment to date, this may reflect different timescales as to how long the patients have suffered their symptoms, and a considerable percentage of the “no treatment” group may go on in the future to have treatment. It may also reflect greater intellectual curiosity and hence longer timescale to consider all options before proceeding to therapeutic procedures. Preliminary evidence suggests that pars plana vitrectomy [6, 21] and Nd:YAG laser treatment improve mental health and quality of life in vitreous floaters patients [6]. Our study does not address this due to the very small numbers within our sample having had surgical or laser treatment.

The main strengths of this study include: 1) one of the few studies examining personality traits and mental health outcomes in a sample of patients with vitreous floaters; 2) the novelty of findings considering the lack of evidence on the topic and its potential relevance for clinical practice; 3) a relatively large sample size compared to previous studies [6]. Our main findings should be interpreted with caution due to the following study limitations: 1) possible sampling bias due to relatively small and not representative sample of patients with floaters, and also due to the fact that our sample characteristics have not been matched with U.K. normative data for sex (unequal proportions), which limits the generalizability of our findings; 2) the fact that our sample has been recruited online mainly due to the limitations imposed by the SARS-COV-2 pandemic in the U.K., might have caused sampling bias due to enrolment, which also limits the generalizability of our findings; 3) the recruitment was undertaken from self-selection and may be biased towards more technologically web based platforms; 4) the cross-sectional design which did not allow us to capture possible variations in mental health outcomes over time; 5) lack of control group (people without any symptomatic floaters); 6) unbalanced patient sub-groups for medical treatment for vitreous floaters and also for gender, which limits generalizability and comparison with normative data; 7) lack of information on patients’ comorbidities which could influence their mental health outcomes; and 8) the scarce literature addressing personality traits in eye disease patients, including vitreous floaters, which makes the comparability of our results more difficult; and 9) using the assumption that a time period of > 3 months duration of symptoms requiring a consultation was a reasonable surrogate measure of severity of symptoms.

In summary, our study highlights the presence of higher levels of neuroticism and lower levels of extraversion personality traits, as well as high prevalence of moderate to severe symptoms of anxiety and depression in a sample of adults with vitreous floaters. Future large-scale and longitudinal studies will clarify the actual importance of these personality traits in vitreous floaters and why these patients may present with mental health comorbidities. The fact that some patients with vitreous floaters are greatly impacted by their symptoms such that they seek medical help and potentially invasive treatments despite not having a vision-threatening condition may be partially explained by their personality traits making accommodation to their new symptoms more difficult to manage and hence greatly impacting their quality of life. In addition, the higher neuroticism traits identified in this study may partly explain patients’ behaviours and their susceptibility to depression and anxiety as suggested in previous studies. A better psychological characterization of this patient group is, therefore, needed, as it will inform clinicians on how to manage patients’ behaviours and expectations in relation to vitreous floaters prognosis and treatment.

## Supplementary Information

Below is the link to the electronic supplementary material.

Supplementary file1 (DOCX 62 KB)
