# Supplementary material for: Personality traits and symptoms of anxiety and depression in patients with primary vitreous floaters
Source: Graefes Arch Clin Exp Ophthalmol. 2024 May 3;262(10):3153–60. doi: 10.1007/s00417-024-06477-y (PMC11458637; doi:10.1007/s00417-024-06477-y)

**SUPPLEMENTARY MATERIALS**

|  | | **Study Sample** | | **UK Normative Data^1^** | |
| --- | --- | --- | --- | --- | --- |
|  |  | N | % | N | % |
| Gender | Male | 108 | 72.5% | 138 820 | 36% |
|  | Female | 41 | 27.5% | 247 551 | 64% |
| Age Groups | 19 or younger | 3 | 2% | 36 700 | 10% |
|  | 20 to 29 | 54 | 36% | 116 262 | 30% |
|  | 30 to 39 | 55 | 37% | 90 059 | 23% |
|  | 40 to 49 | 18 | 12% | 73 393 | 19% |
|  | 50 to 59 | 10 | 7% | 44 218 | 11% |
|  | 60 to 69 | 7 | 5% | 21 407 | 6% |
|  | 70 and older | 2 | 1% | 4 336 | 1% |

**Table A** – Sample and UK normative data^1^ characteristics. ^1^ Rentfrow PJ, Jokela M, Lamb ME (2015) Regional personality differences in Great Britain. PLoS One 10:. <https://doi.org/10.1371/journal.pone.0122245>

1. **Normality tests to BFI outcome measures**

| BFI Personality Trait | Shapiro-Wilk Test  (W; p-value) |
| --- | --- |
| Neuroticism | W = 0.98; p = 0.08 |
| Extraversion | W = 0.99; p = 0.34 |
| Conscientiousness | W = 0.98; p = 0.11 |
| Agreeableness | W = 0.98; p = 0.10 |
| Openness | W = 0.99; p = 0.14 |

1. **Histograms**


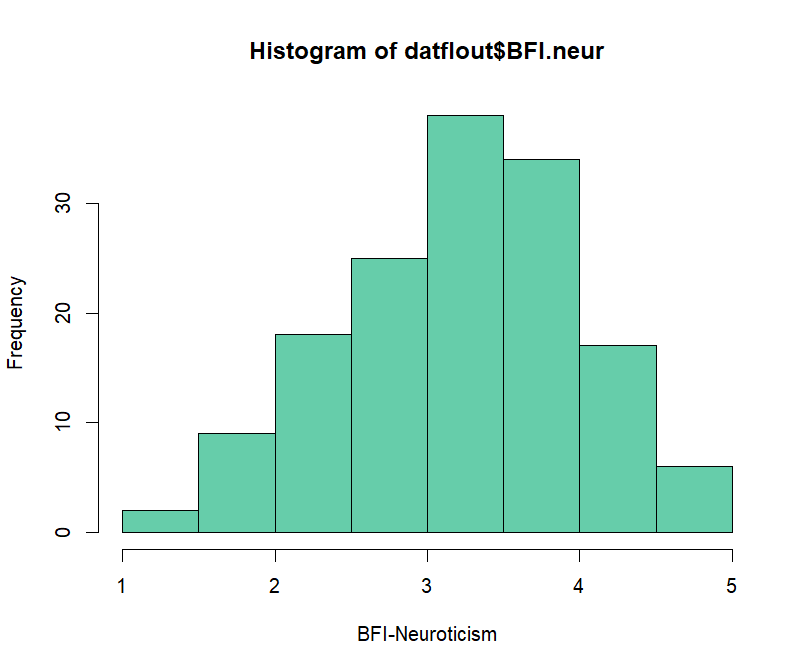


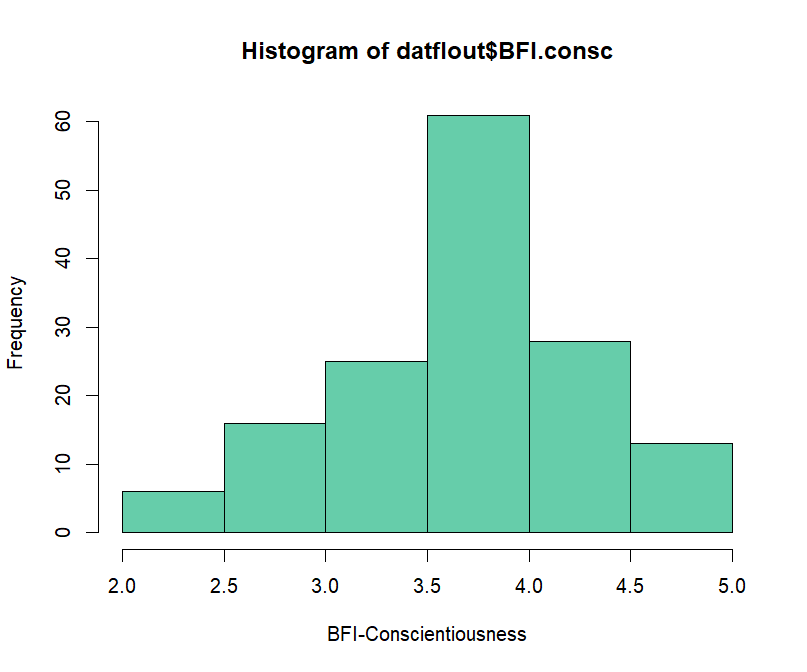


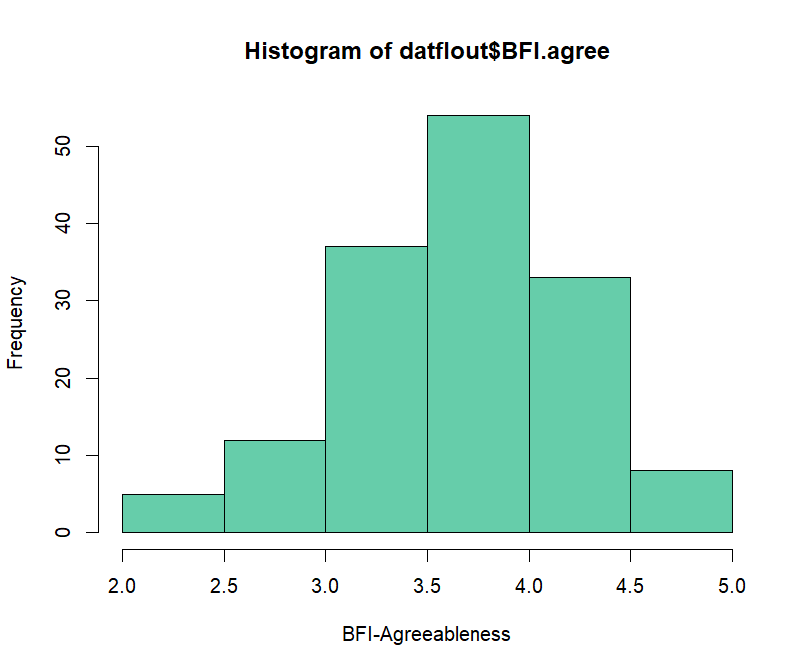


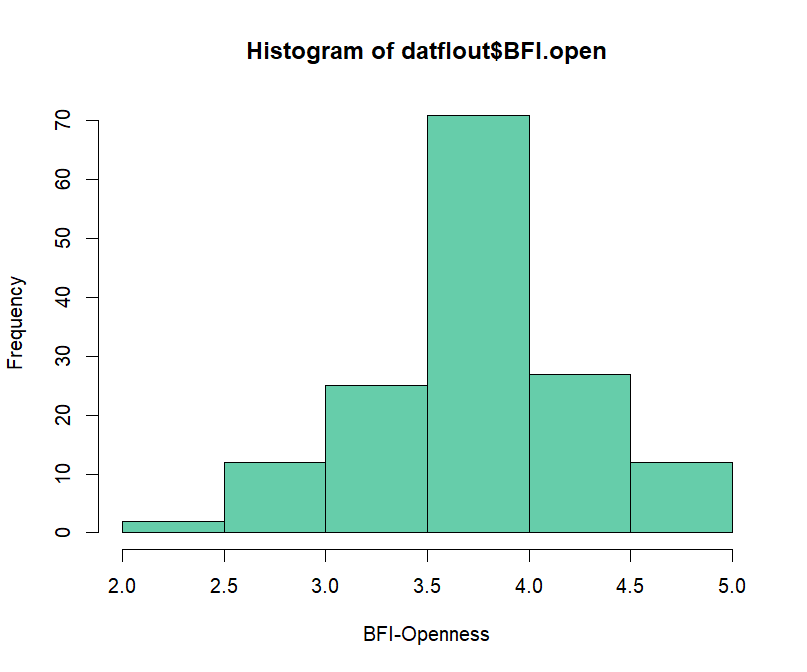


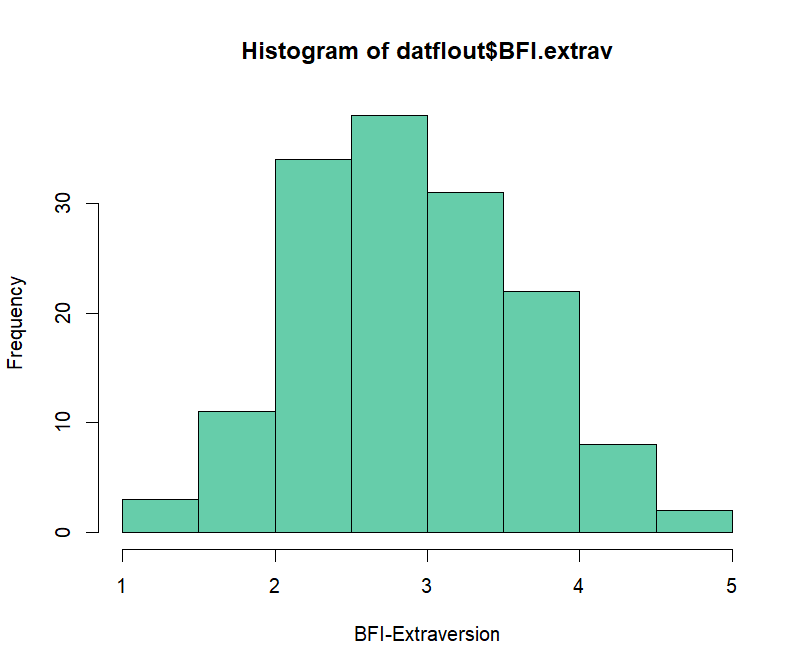

Supplement: Supplementary file 1 — Supplementary file1 (DOCX 62 KB) [file 417_2024_6477_MOESM1_ESM.docx]
